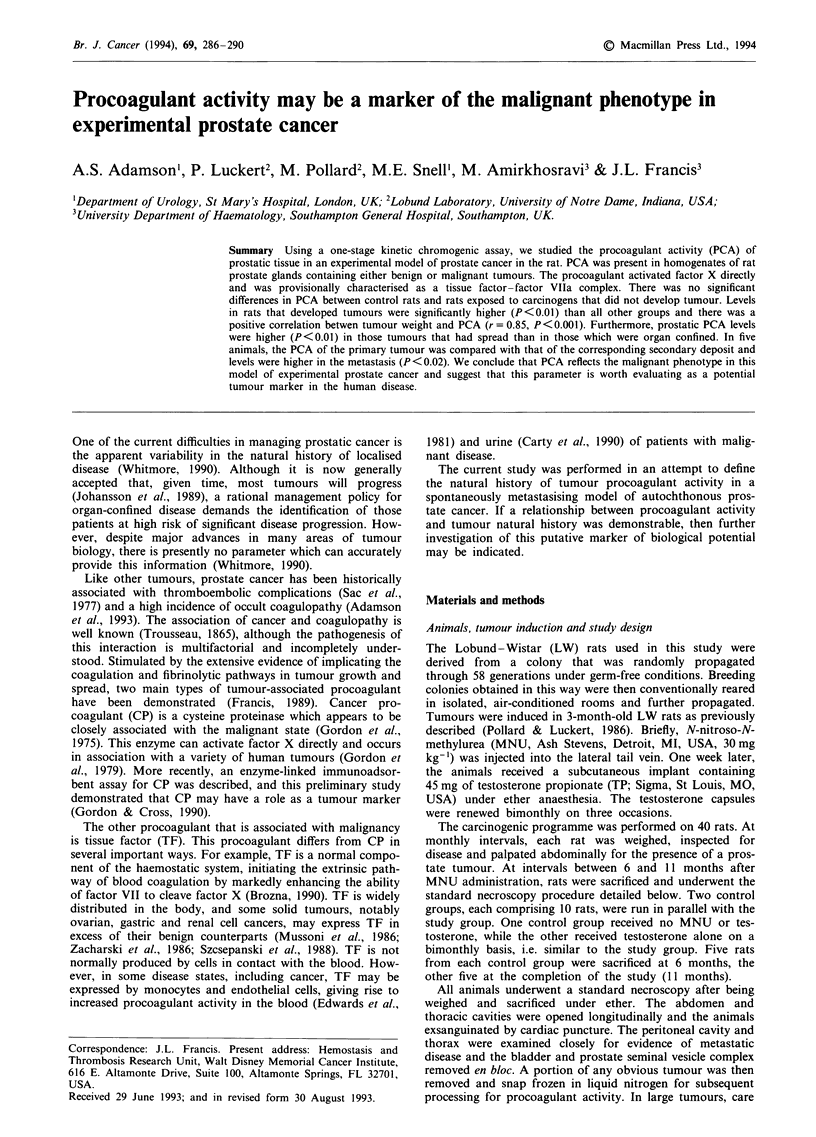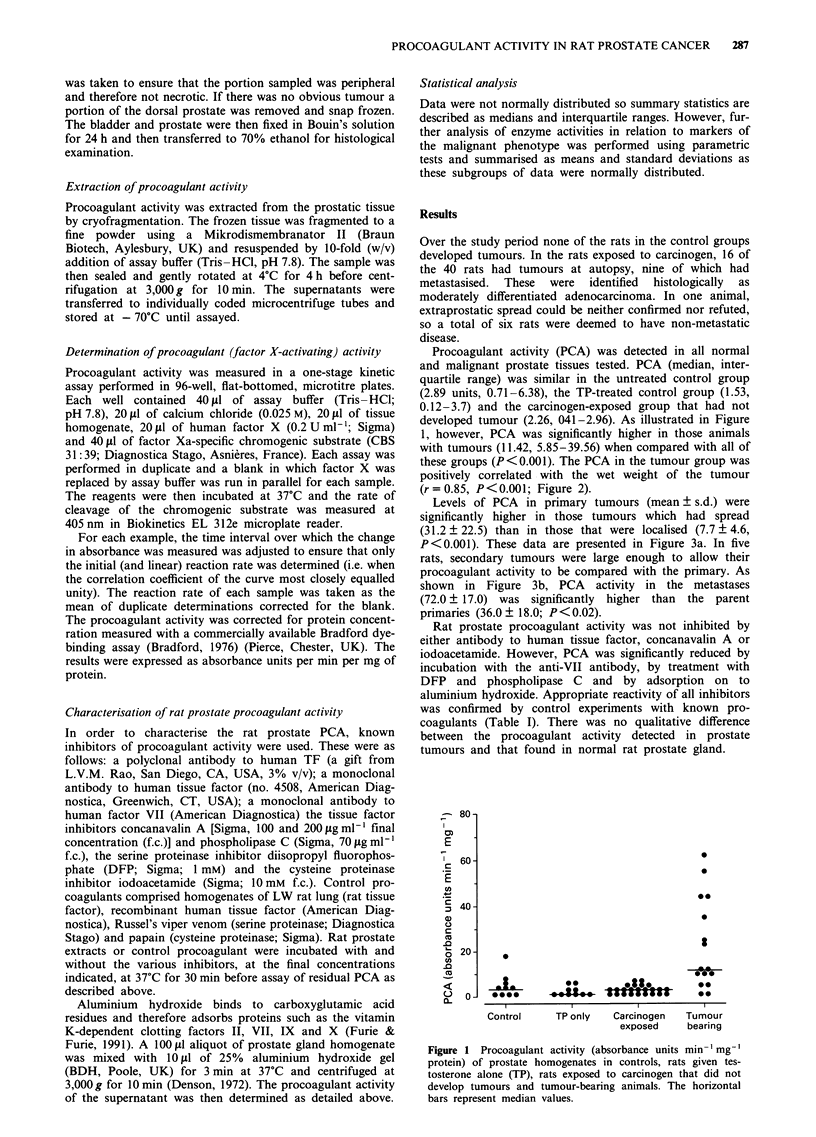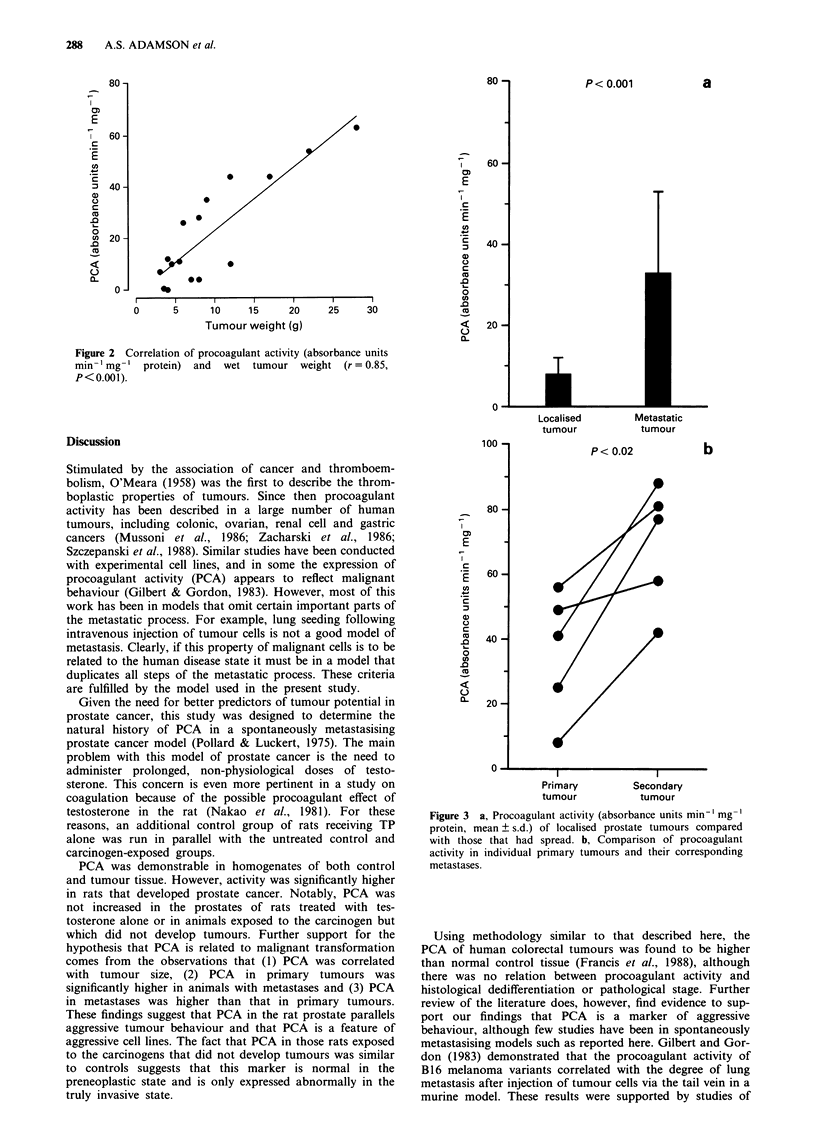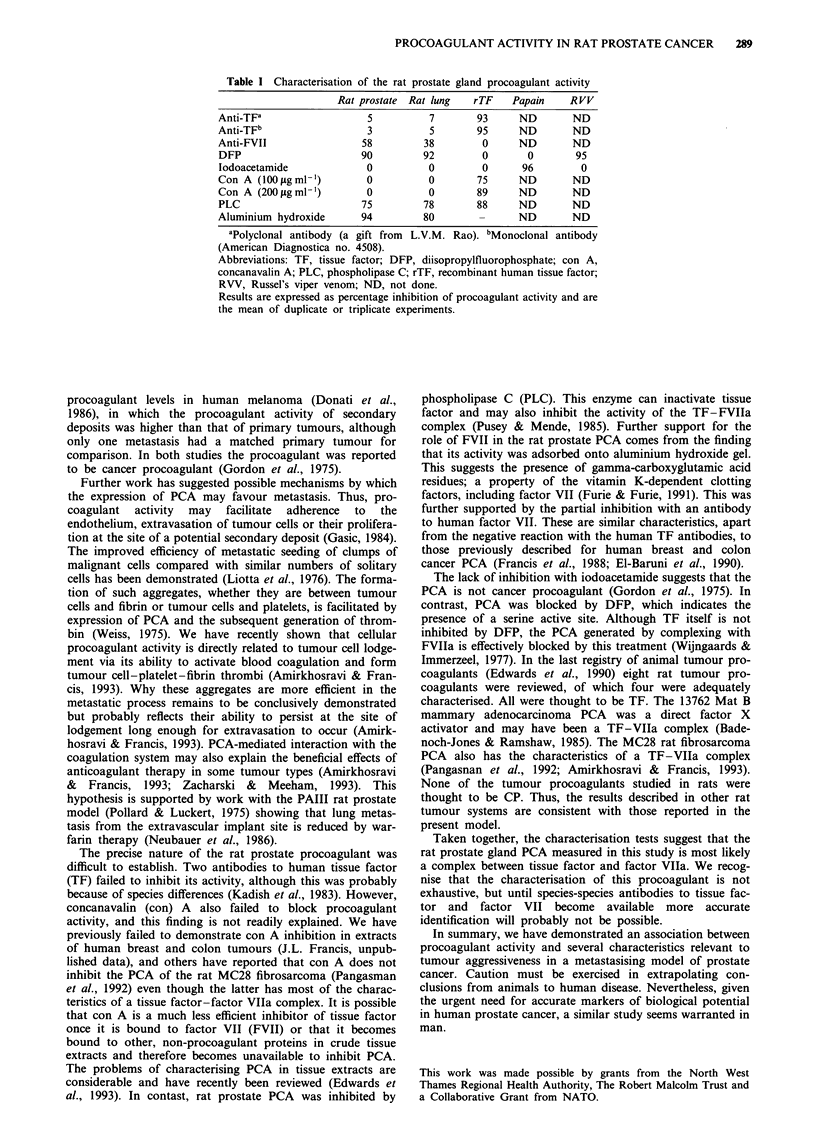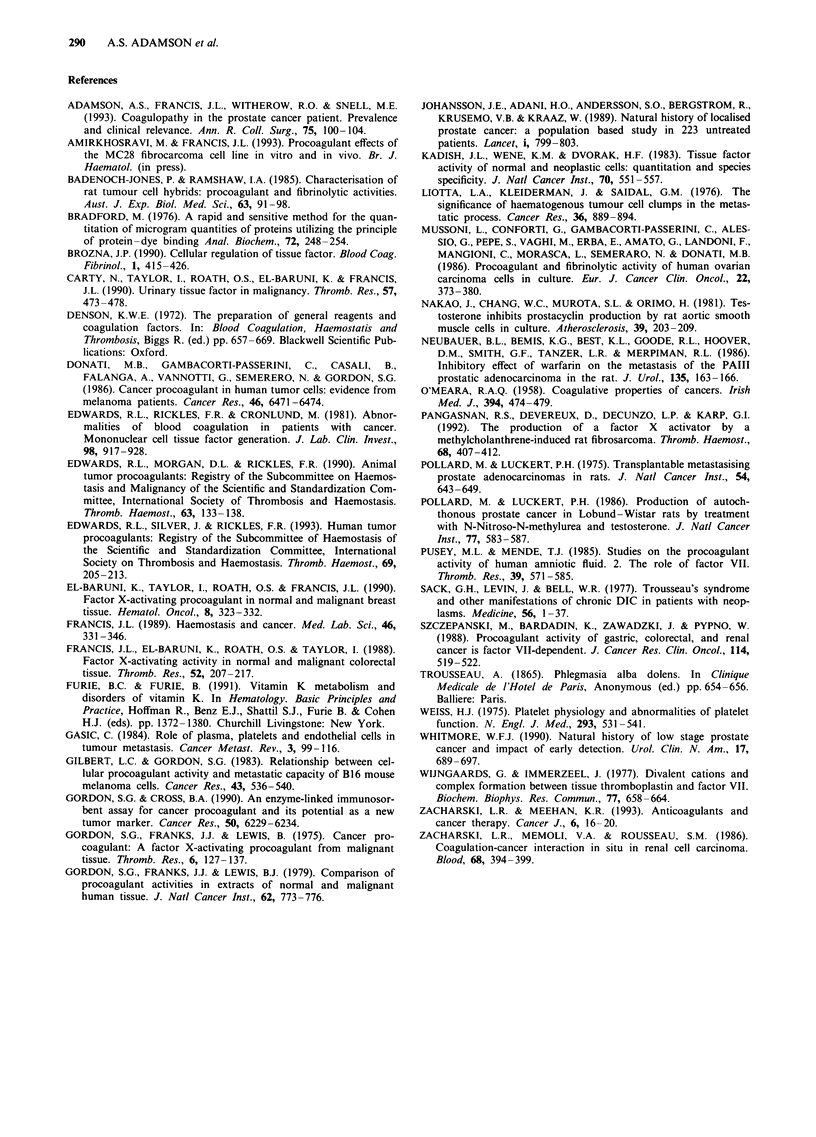# Procoagulant activity may be a marker of the malignant phenotype in experimental prostate cancer.

**DOI:** 10.1038/bjc.1994.53

**Published:** 1994-02

**Authors:** A. S. Adamson, P. Luckert, M. Pollard, M. E. Snell, M. Amirkhosravi, J. L. Francis

**Affiliations:** Department of Urology, St Mary's Hospital, London, UK.

## Abstract

Using a one-stage kinetic chromogenic assay, we studied the procoagulant activity (PCA) of prostatic tissue in an experimental model of prostate cancer in the rat. PCA was present in homogenates of rat prostate glands containing either benign or malignant tumours. The procoagulant activated factor X directly and was provisionally characterised as a tissue factor-factor VIIa complex. There was no significant differences in PCA between control rats and rats exposed to carcinogens that did not develop tumour. Levels in rats that developed tumours were significantly higher (P < 0.01) than all other groups and there was a positive correlation between tumour weight and PCA (r = 0.85, P < 0.001). Furthermore, prostatic PCA levels were higher in the metastasis (P < 0.02). We conclude that PCA reflects the malignant phenotype in this animals, the PCA of the primary tumour was compared with that of the corresponding secondary deposit and levels were higher in the metastasis (P < 0.02). We conclude that PCA reflects the malignant phenotype in this model of experimental prostate cancer and suggest that this parameter is worth evaluating as a potential tumour marker in the human disease.


Br. J. Cancer (1994), 69, 286-290 © Macmillan Press Ltd., 1994

### Procoagulant activity may be a marker of the malignant phenotype in experimental prostate cancer

A.S. Adamson[1], P. Luckert[2], M. Pollard[2], M.E. Snell[1], M. Amirkhosravi[3] & J.L. Francis[3]

[1]*Department of Urology, St Mary's Hospital, London, UK;* [2]*Lobund Laboratory, University of Notre Dame, Indiana, USA;* [3]*University Department of Haematology, Southampton General Hospital, Southampton, UK.*

**Summary** Using a one-stage kinetic chromogenic assay, we studied the procoagulant activity (PCA) of prostatic tissue in an experimental model of prostate cancer in the rat. PCA was present in homogenates of rat prostate glands containing either benign or malignant tumours. The procoagulant activated factor X directly and was provisionally characterised as a tissue factor–factor VIIa complex. There was no significant differences in PCA between control rats and rats exposed to carcinogens that did not develop tumour. Levels in rats that developed tumours were significantly higher ($P<0.01$) than all other groups and there was a positive correlation betwen tumour weight and PCA ($r = 0.85$, $P<0.001$). Furthermore, prostatic PCA levels were higher ($P<0.01$) in those tumours that had spread than in those which were organ confined. In five animals, the PCA of the primary tumour was compared with that of the corresponding secondary deposit and levels were higher in the metastasis ($P<0.02$). We conclude that PCA reflects the malignant phenotype in this model of experimental prostate cancer and suggest that this parameter is worth evaluating as a potential tumour marker in the human disease.

One of the current difficulties in managing prostatic cancer is the apparent variability in the natural history of localised disease (Whitmore, 1990). Although it is now generally accepted that, given time, most tumours will progress (Johansson *et al.*, 1989), a rational management policy for organ-confined disease demands the identification of those patients at high risk of significant disease progression. However, despite major advances in many areas of tumour biology, there is presently no parameter which can accurately provide this information (Whitmore, 1990).

Like other tumours, prostate cancer has been historically associated with thromboembolic complications (Sac *et al.*, 1977) and a high incidence of occult coagulopathy (Adamson *et al.*, 1993). The association of cancer and coagulopathy is well known (Trousseau, 1865), although the pathogenesis of this interaction is multifactorial and incompletely understood. Stimulated by the extensive evidence of implicating the coagulation and fibrinolytic pathways in tumour growth and spread, two main types of tumour-associated procoagulant have been demonstrated (Francis, 1989). Cancer procoagulant (CP) is a cysteine proteinase which appears to be closely associated with the malignant state (Gordon *et al.*, 1975). This enzyme can activate factor X directly and occurs in association with a variety of human tumours (Gordon *et al.*, 1979). More recently, an enzyme-linked immunoadsorbent assay for CP was described, and this preliminary study demonstrated that CP may have a role as a tumour marker (Gordon & Cross, 1990).

The other procoagulant that is associated with malignancy is tissue factor (TF). This procoagulant differs from CP in several important ways. For example, TF is a normal component of the haemostatic system, initiating the extrinsic pathway of blood coagulation by markedly enhancing the ability of factor VII to cleave factor X (Brozna, 1990). TF is widely distributed in the body, and some solid tumours, notably ovarian, gastric and renal cell cancers, may express TF in excess of their benign counterparts (Mussoni *et al.*, 1986; Zacharski *et al.*, 1986; Szcsepanski *et al.*, 1988). TF is not normally produced by cells in contact with the blood. However, in some disease states, including cancer, TF may be expressed by monocytes and endothelial cells, giving rise to increased procoagulant activity in the blood (Edwards *et al.*, 1981) and urine (Carty *et al.*, 1990) of patients with malignant disease.

The current study was performed in an attempt to define the natural history of tumour procoagulant activity in a spontaneously metastasising model of autochthonous prostate cancer. If a relationship between procoagulant activity and tumour natural history was demonstrable, then further investigation of this putative marker of biological potential may be indicated.

### Materials and methods

### *Animals, tumour induction and study design*

The Lobund–Wistar (LW) rats used in this study were derived from a colony that was randomly propagated through 58 generations under germ-free conditions. Breeding colonies obtained in this way were then conventionally reared in isolated, air-conditioned rooms and further propagated. Tumours were induced in 3-month-old LW rats as previously described (Pollard & Luckert, 1986). Briefly, *N*-nitroso-*N*-methylurea (MNU, Ash Stevens, Detroit, MI, USA, 30 mg $kg^{-1}$) was injected into the lateral tail vein. One week later, the animals received a subcutaneous implant containing 45 mg of testosterone propionate (TP; Sigma, St Louis, MO, USA) under ether anaesthesia. The testosterone capsules were renewed bimonthly on three occasions.

The carcinogenic programme was performed on 40 rats. At monthly intervals, each rat was weighed, inspected for disease and palpated abdominally for the presence of a prostate tumour. At intervals between 6 and 11 months after MNU administration, rats were sacrificed and underwent the standard necroscopy procedure detailed below. Two control groups, each comprising 10 rats, were run in parallel with the study group. One control group received no MNU or testosterone, while the other received testosterone alone on a bimonthly basis, i.e. similar to the study group. Five rats from each control group were sacrificed at 6 months, the other five at the completion of the study (11 months).

All animals underwent a standard necroscopy after being weighed and sacrificed under ether. The abdomen and thoracic cavities were opened longitudinally and the animals exsanguinated by cardiac puncture. The peritoneal cavity and thorax were examined closely for evidence of metastatic disease and the bladder and prostate seminal vesicle complex removed *en bloc*. A portion of any obvious tumour was then removed and snap frozen in liquid nitrogen for subsequent processing for procoagulant activity. In large tumours, care

Correspondence: J.L. Francis. Present address: Hemostasis and Thrombosis Research Unit, Walt Disney Memorial Cancer Institute, 616 E. Altamonte Drive, Suite 100, Altamonte Springs, FL 32701, USA.
Received 29 June 1993; and in revised form 30 August 1993.

was taken to ensure that the portion sampled was peripheral and therefore not necrotic. If there was no obvious tumour a portion of the dorsal prostate was removed and snap frozen. The bladder and prostate were then fixed in Bouin's solution for 24 h and then transferred to 70% ethanol for histological examination.

*Extraction of procoagulant activity*

Procoagulant activity was extracted from the prostatic tissue by cryofragmentation. The frozen tissue was fragmented to a fine powder using a Mikrodismembranator II (Braun Biotech, Aylesbury, UK) and resuspended by 10-fold (w/v) addition of assay buffer (Tris-HCl, pH 7.8). The sample was then sealed and gently rotated at 4°C for 4 h before centrifugation at 3,000 *g* for 10 min. The supernatants were transferred to individually coded microcentrifuge tubes and stored at − 70°C until assayed.

*Determination of procoagulant (factor X-activating) activity*

Procoagulant activity was measured in a one-stage kinetic assay performed in 96-well, flat-bottomed, microtitre plates. Each well contained 40 µl of assay buffer (Tris-HCl; pH 7.8), 20 µl of calcium chloride (0.025 M), 20 µl of tissue homogenate, 20 µl of human factor X (0.2 U $ml^{-1}$; Sigma) and 40 µl of factor Xa-specific chromogenic substrate (CBS 31:39; Diagnostica Stago, Asnières, France). Each assay was performed in duplicate and a blank in which factor X was replaced by assay buffer was run in parallel for each sample. The reagents were then incubated at 37°C and the rate of cleavage of the chromogenic substrate was measured at 405 nm in Biokinetics EL 312e microplate reader.

For each example, the time interval over which the change in absorbance was measured was adjusted to ensure that only the initial (and linear) reaction rate was determined (i.e. when the correlation coefficient of the curve most closely equalled unity). The reaction rate of each sample was taken as the mean of duplicate determinations corrected for the blank. The procoagulant activity was corrected for protein concentration measured with a commercially available Bradford dye-binding assay (Bradford, 1976) (Pierce, Chester, UK). The results were expressed as absorbance units per min per mg of protein.

*Characterisation of rat prostate procoagulant activity*

In order to characterise the rat prostate PCA, known inhibitors of procoagulant activity were used. These were as follows: a polyclonal antibody to human TF (a gift from L.V.M. Rao, San Diego, CA, USA, 3% v/v); a monoclonal antibody to human tissue factor (no. 4508, American Diagnostica, Greenwich, CT, USA); a monoclonal antibody to human factor VII (American Diagnostica) the tissue factor inhibitors concanavalin A [Sigma, 100 and 200 µg $ml^{-1}$ final concentration (f.c.)] and phospholipase C (Sigma, 70 µg $ml^{-1}$ f.c.), the serine proteinase inhibitor diisopropyl fluorophosphate (DFP; Sigma; 1 mM) and the cysteine proteinase inhibitor iodoacetamide (Sigma; 10 mM f.c.). Control procoagulants comprised homogenates of LW rat lung (rat tissue factor), recombinant human tissue factor (American Diagnostica), Russel's viper venom (serine proteinase; Diagnostica Stago) and papain (cysteine proteinase; Sigma). Rat prostate extracts or control procoagulant were incubated with and without the various inhibitors, at the final concentrations indicated, at 37°C for 30 min before assay of residual PCA as described above.

Aluminium hydroxide binds to carboxyglutamic acid residues and therefore adsorbs proteins such as the vitamin K-dependent clotting factors II, VII, IX and X (Furie & Furie, 1991). A 100 µl aliquot of prostate gland homogenate was mixed with 10 µl of 25% aluminium hydroxide gel (BDH, Poole, UK) for 3 min at 37°C and centrifuged at 3,000 *g* for 10 min (Denson, 1972). The procoagulant activity of the supernatant was then determined as detailed above.

*Statistical analysis*

Data were not normally distributed so summary statistics are described as medians and interquartile ranges. However, further analysis of enzyme activities in relation to markers of the malignant phenotype was performed using parametric tests and summarised as means and standard deviations as these subgroups of data were normally distributed.

### Results

Over the study period none of the rats in the control groups developed tumours. In the rats exposed to carcinogen, 16 of the 40 rats had tumours at autopsy, nine of which had metastasised. These were identified histologically as moderately differentiated adenocarcinoma. In one animal, extraprostatic spread could be neither confirmed nor refuted, so a total of six rats were deemed to have non-metastatic disease.

Procoagulant activity (PCA) was detected in all normal and malignant prostate tissues tested. PCA (median, interquartile range) was similar in the untreated control group (2.89 units, 0.71–6.38), the TP-treated control group (1.53, 0.12–3.7) and the carcinogen-exposed group that had not developed tumour (2.26, 041–2.96). As illustrated in Figure 1, however, PCA was significantly higher in those animals with tumours (11.42, 5.85–39.56) when compared with all of these groups ($P < 0.001$). The PCA in the tumour group was positively correlated with the wet weight of the tumour ($r = 0.85$, $P < 0.001$; Figure 2).

Levels of PCA in primary tumours (mean ± s.d.) were significantly higher in those tumours which had spread (31.2 ± 22.5) than in those that were localised (7.7 ± 4.6, $P < 0.001$). These data are presented in Figure 3a. In five rats, secondary tumours were large enough to allow their procoagulant activity to be compared with the primary. As shown in Figure 3b, PCA activity in the metastases (72.0 ± 17.0) was significantly higher than the parent primaries (36.0 ± 18.0; $P < 0.02$).

Rat prostate procoagulant activity was not inhibited by either antibody to human tissue factor, concanavalin A or iodoacetamide. However, PCA was significantly reduced by incubation with the anti-VII antibody, by treatment with DFP and phospholipase C and by adsorption on to aluminium hydroxide. Appropriate reactivity of all inhibitors was confirmed by control experiments with known procoagulants (Table I). There was no qualitative difference between the procoagulant activity detected in prostate tumours and that found in normal rat prostate gland.

**Figure 1** Procoagulant activity (absorbance units $min^{-1}$ $mg^{-1}$ protein) of prostate homogenates in controls, rats given testosterone alone (TP), rats exposed to carcinogen that did not develop tumours and tumour-bearing animals. The horizontal bars represent median values.

**Figure 2** Correlation of procoagulant activity (absorbance units $min^{-1}mg^{-1}$ protein) and wet tumour weight ($r = 0.85$, $P < 0.001$).

**Figure 3** **a**, Procoagulant activity (absorbance units $min^{-1}$ $mg^{-1}$ protein, mean ± s.d.) of localised prostate tumours compared with those that had spread. **b**, Comparison of procoagulant activity in individual primary tumours and their corresponding metastases.

### Discussion

Stimulated by the association of cancer and thromboembolism, O'Meara (1958) was the first to describe the thromboplastic properties of tumours. Since then procoagulant activity has been described in a large number of human tumours, including colonic, ovarian, renal cell and gastric cancers (Mussoni *et al.*, 1986; Zacharski *et al.*, 1986; Szczepanski *et al.*, 1988). Similar studies have been conducted with experimental cell lines, and in some the expression of procoagulant activity (PCA) appears to reflect malignant behaviour (Gilbert & Gordon, 1983). However, most of this work has been in models that omit certain important parts of the metastatic process. For example, lung seeding following intravenous injection of tumour cells is not a good model of metastasis. Clearly, if this property of malignant cells is to be related to the human disease state it must be in a model that duplicates all steps of the metastatic process. These criteria are fulfilled by the model used in the present study.

Given the need for better predictors of tumour potential in prostate cancer, this study was designed to determine the natural history of PCA in a spontaneously metastasising prostate cancer model (Pollard & Luckert, 1975). The main problem with this model of prostate cancer is the need to administer prolonged, non-physiological doses of testosterone. This concern is even more pertinent in a study on coagulation because of the possible procoagulant effect of testosterone in the rat (Nakao *et al.*, 1981). For these reasons, an additional control group of rats receiving TP alone was run in parallel with the untreated control and carcinogen-exposed groups.

PCA was demonstrable in homogenates of both control and tumour tissue. However, activity was significantly higher in rats that developed prostate cancer. Notably, PCA was not increased in the prostates of rats treated with testosterone alone or in animals exposed to the carcinogen but which did not develop tumours. Further support for the hypothesis that PCA is related to malignant transformation comes from the observations that (1) PCA was correlated with tumour size, (2) PCA in primary tumours was significantly higher in animals with metastases and (3) PCA in metastases was higher than that in primary tumours. These findings suggest that PCA in the rat prostate parallels aggressive tumour behaviour and that PCA is a feature of aggressive cell lines. The fact that PCA in those rats exposed to the carcinogens that did not develop tumours was similar to controls suggests that this marker is normal in the preneoplastic state and is only expressed abnormally in the truly invasive state.

Using methodology similar to that described here, the PCA of human colorectal tumours was found to be higher than normal control tissue (Francis *et al.*, 1988), although there was no relation between procoagulant activity and histological dedifferentiation or pathological stage. Further review of the literature does, however, find evidence to support our findings that PCA is a marker of aggressive behaviour, although few studies have been in spontaneously metastasising models such as reported here. Gilbert and Gordon (1983) demonstrated that the procoagulant activity of B16 melanoma variants correlated with the degree of lung metastasis after injection of tumour cells via the tail vein in a murine model. These results were supported by studies of

**Table I** Characterisation of the rat prostate gland procoagulant activity

| | *Rat prostate* | *Rat lung* | *rTF* | *Papain* | *RVV* |
|---|---|---|---|---|---|
| Anti-TF[a] | 5 | 7 | 93 | ND | ND |
| Anti-TF[b] | 3 | 5 | 95 | ND | ND |
| Anti-FVII | 58 | 38 | 0 | ND | ND |
| DFP | 90 | 92 | 0 | 0 | 95 |
| Iodoacetamide | 0 | 0 | 0 | 96 | 0 |
| Con A (100 $\mu g\ ml^{-1}$) | 0 | 0 | 75 | ND | ND |
| Con A (200 $\mu g\ ml^{-1}$) | 0 | 0 | 89 | ND | ND |
| PLC | 75 | 78 | 88 | ND | ND |
| Aluminium hydroxide | 94 | 80 | – | ND | ND |

[a]Polyclonal antibody (a gift from L.V.M. Rao). [b]Monoclonal antibody (American Diagnostica no. 4508).
Abbreviations: TF, tissue factor; DFP, diisopropylfluorophosphate; con A, concanavalin A; PLC, phospholipase C; rTF, recombinant human tissue factor; RVV, Russel's viper venom; ND, not done.
Results are expressed as percentage inhibition of procoagulant activity and are the mean of duplicate or triplicate experiments.

procoagulant levels in human melanoma (Donati *et al.*, 1986), in which the procoagulant activity of secondary deposits was higher than that of primary tumours, although only one metastasis had a matched primary tumour for comparison. In both studies the procoagulant was reported to be cancer procoagulant (Gordon *et al.*, 1975).

Further work has suggested possible mechanisms by which the expression of PCA may favour metastasis. Thus, procoagulant activity may facilitate adherence to the endothelium, extravasation of tumour cells or their proliferation at the site of a potential secondary deposit (Gasic, 1984). The improved efficiency of metastatic seeding of clumps of malignant cells compared with similar numbers of solitary cells has been demonstrated (Liotta *et al.*, 1976). The formation of such aggregates, whether they are between tumour cells and fibrin or tumour cells and platelets, is facilitated by expression of PCA and the subsequent generation of thrombin (Weiss, 1975). We have recently shown that cellular procoagulant activity is directly related to tumour cell lodgement via its ability to activate blood coagulation and form tumour cell–platelet–fibrin thrombi (Amirkhosravi & Francis, 1993). Why these aggregates are more efficient in the metastatic process remains to be conclusively demonstrated but probably reflects their ability to persist at the site of lodgement long enough for extravasation to occur (Amirkhosravi & Francis, 1993). PCA-mediated interaction with the coagulation system may also explain the beneficial effects of anticoagulant therapy in some tumour types (Amirkhosravi & Francis, 1993; Zacharski & Meeham, 1993). This hypothesis is supported by work with the PAIII rat prostate model (Pollard & Luckert, 1975) showing that lung metastasis from the extravascular implant site is reduced by warfarin therapy (Neubauer *et al.*, 1986).

The precise nature of the rat prostate procoagulant was difficult to establish. Two antibodies to human tissue factor (TF) failed to inhibit its activity, although this was probably because of species differences (Kadish *et al.*, 1983). However, concanavalin (con) A also failed to block procoagulant activity, and this finding is not readily explained. We have previously failed to demonstrate con A inhibition in extracts of human breast and colon tumours (J.L. Francis, unpublished data), and others have reported that con A does not inhibit the PCA of the rat MC28 fibrosarcoma (Pangasman *et al.*, 1992) even though the latter has most of the characteristics of a tissue factor–factor VIIa complex. It is possible that con A is a much less efficient inhibitor of tissue factor once it is bound to factor VII (FVII) or that it becomes bound to other, non-procoagulant proteins in crude tissue extracts and therefore becomes unavailable to inhibit PCA. The problems of characterising PCA in tissue extracts are considerable and have recently been reviewed (Edwards *et al.*, 1993). In contast, rat prostate PCA was inhibited by phospholipase C (PLC). This enzyme can inactivate tissue factor and may also inhibit the activity of the TF–FVIIa complex (Pusey & Mende, 1985). Further support for the role of FVII in the rat prostate PCA comes from the finding that its activity was adsorbed onto aluminium hydroxide gel. This suggests the presence of gamma-carboxyglutamic acid residues; a property of the vitamin K-dependent clotting factors, including factor VII (Furie & Furie, 1991). This was further supported by the partial inhibition with an antibody to human factor VII. These are similar characteristics, apart from the negative reaction with the human TF antibodies, to those previously described for human breast and colon cancer PCA (Francis *et al.*, 1988; El-Baruni *et al.*, 1990).

The lack of inhibition with iodoacetamide suggests that the PCA is not cancer procoagulant (Gordon *et al.*, 1975). In contrast, PCA was blocked by DFP, which indicates the presence of a serine active site. Although TF itself is not inhibited by DFP, the PCA generated by complexing with FVIIa is effectively blocked by this treatment (Wijngaards & Immerzeel, 1977). In the last registry of animal tumour procoagulants (Edwards *et al.*, 1990) eight rat tumour procoagulants were reviewed, of which four were adequately characterised. All were thought to be TF. The 13762 Mat B mammary adenocarcinoma PCA was a direct factor X activator and may have been a TF–VIIa complex (Badenoch-Jones & Ramshaw, 1985). The MC28 rat fibrosarcoma PCA also has the characteristics of a TF–VIIa complex (Pangasnan *et al.*, 1992; Amirkhosravi & Francis, 1993). None of the tumour procoagulants studied in rats were thought to be CP. Thus, the results described in other rat tumour systems are consistent with those reported in the present model.

Taken together, the characterisation tests suggest that the rat prostate gland PCA measured in this study is most likely a complex between tissue factor and factor VIIa. We recognise that the characterisation of this procoagulant is not exhaustive, but until species-species antibodies to tissue factor and factor VII become available more accurate identification will probably not be possible.

In summary, we have demonstrated an association between procoagulant activity and several characteristics relevant to tumour aggressiveness in a metastasising model of prostate cancer. Caution must be exercised in extrapolating conclusions from animals to human disease. Nevertheless, given the urgent need for accurate markers of biological potential in human prostate cancer, a similar study seems warranted in man.

This work was made possible by grants from the North West Thames Regional Health Authority, The Robert Malcolm Trust and a Collaborative Grant from NATO.

### References

ADAMSON, A.S., FRANCIS, J.L., WITHEROW, R.O. & SNELL, M.E. (1993). Coagulopathy in the prostate cancer patient. Prevalence and clinical relevance. *Ann. R. Coll. Surg.*, **75**, 100–104.

AMIRKHOSRAVI, M. & FRANCIS, J.L. (1993). Procoagulant effects of the MC28 fibrocarcoma cell line in vitro and in vivo. *Br. J. Haematol.* (in press).

BADENOCH-JONES, P. & RAMSHAW, I.A. (1985). Characterisation of rat tumour cell hybrids: procoagulant and fibrinolytic activities. *Aust. J. Exp. Biol. Med. Sci.*, **63**, 91–98.

BRADFORD, M. (1976). A rapid and sensitive method for the quantitation of microgram quantities of proteins utilizing the principle of protein–dye binding *Anal. Biochem.*, **72**, 248–254.

BROZNA, J.P. (1990). Cellular regulation of tissue factor. *Blood Coag. Fibrinol.*, **1**, 415–426.

CARTY, N., TAYLOR, I., ROATH, O.S., EL-BARUNI, K. & FRANCIS, J.L. (1990). Urinary tissue factor in malignancy. *Thromb. Res.*, **57**, 473–478.

DENSON, K.W.E. (1972). The preparation of general reagents and coagulation factors. In: *Blood Coagulation, Haemostatis and Thrombosis*, Biggs R. (ed.) pp. 657–669. Blackwell Scientific Publications: Oxford.

DONATI, M.B., GAMBACORTI-PASSERINI, C., CASALI, B., FALANGA, A., VANNOTTI, G., SEMERERO, N. & GORDON, S.G. (1986). Cancer procoagulant in human tumor cells: evidence from melanoma patients. *Cancer Res.*, **46**, 6471–6474.

EDWARDS, R.L., RICKLES, F.R. & CRONLUND, M. (1981). Abnormalities of blood coagulation in patients with cancer. Mononuclear cell tissue factor generation. *J. Lab. Clin. Invest.*, **98**, 917–928.

EDWARDS, R.L., MORGAN, D.L. & RICKLES, F.R. (1990). Animal tumor procoagulants: Registry of the Subcommittee on Haemostasis and Malignancy of the Scientific and Standardization Committee, International Society of Thrombosis and Haemostasis. *Thromb. Haemost.*, **63**, 133–138.

EDWARDS, R.L., SILVER, J. & RICKLES, F.R. (1993). Human tumor procoagulants: Registry of the Subcommittee of Haemostasis of the Scientific and Standardization Committee, International Society on Thrombosis and Haemostasis. *Thromb. Haemost.*, **69**, 205–213.

EL-BARUNI, K., TAYLOR, I., ROATH, O.S. & FRANCIS, J.L. (1990). Factor X-activating procoagulant in normal and malignant breast tissue. *Hematol. Oncol.*, **8**, 323–332.

FRANCIS, J.L. (1989). Haemostasis and cancer. *Med. Lab. Sci.*, **46**, 331–346.

FRANCIS, J.L., EL-BARUNI, K., ROATH, O.S. & TAYLOR, I. (1988). Factor X-activating activity in normal and malignant colorectal tissue. *Thromb. Res.*, **52**, 207–217.

FURIE, B.C. & FURIE, B. (1991). Vitamin K metabolism and disorders of vitamin K. In *Hematology. Basic Principles and Practice*, Hoffman R., Benz E.J., Shattil S.J., Furie B. & Cohen H.J. (eds). pp. 1372–1380. Churchill Livingstone: New York.

GASIC, C. (1984). Role of plasma, platelets and endothelial cells in tumour metastasis. *Cancer Metast. Rev.*, **3**, 99–116.

GILBERT, L.C. & GORDON, S.G. (1983). Relationship between cellular procoagulant activity and metastatic capacity of B16 mouse melanoma cells. *Cancer Res.*, **43**, 536–540.

GORDON, S.G. & CROSS, B.A. (1990). An enzyme-linked immunosorbent assay for cancer procoagulant and its potential as a new tumor marker. *Cancer Res.*, **50**, 6229–6234.

GORDON, S.G., FRANKS, J.J. & LEWIS, B. (1975). Cancer procoagulant: A factor X-activating procoagulant from malignant tissue. *Thromb. Res.*, **6**, 127–137.

GORDON, S.G., FRANKS, J.J. & LEWIS, B.J. (1979). Comparison of procoagulant activities in extracts of normal and malignant human tissue. *J. Natl Cancer Inst.*, **62**, 773–776.

JOHANSSON, J.E., ADANI, H.O., ANDERSSON, S.O., BERGSTROM, R., KRUSEMO, V.B. & KRAAZ, W. (1989). Natural history of localised prostate cancer: a population based study in 223 untreated patients. *Lancet*, **i**, 799–803.

KADISH, J.L., WENE, K.M. & DVORAK, H.F. (1983). Tissue factor activity of normal and neoplastic cells: quantitation and species specificity. *J. Natl Cancer Inst.*, **70**, 551–557.

LIOTTA, L.A., KLEIDERMAN, J. & SAIDAL, G.M. (1976). The significance of haematogenous tumour cell clumps in the metastatic process. *Cancer Res.*, **36**, 889–894.

MUSSONI, L., CONFORTI, G., GAMBACORTI-PASSERINI, C., ALESSIO, G., PEPE, S., VAGHI, M., ERBA, E., AMATO, G., LANDONI, F., MANGIONI, C., MORASCA, L., SEMERARO, N. & DONATI, M.B. (1986). Procoagulant and fibrinolytic activity of human ovarian carcinoma cells in culture. *Eur. J. Cancer Clin. Oncol.*, **22**, 373–380.

NAKAO, J., CHANG, W.C., MUROTA, S.L. & ORIMO, H. (1981). Testosterone inhibits prostacyclin production by rat aortic smooth muscle cells in culture. *Atherosclerosis*, **39**, 203–209.

NEUBAUER, B.L., BEMIS, K.G., BEST, K.L., GOODE, R.L., HOOVER, D.M., SMITH, G.F., TANZER, L.R. & MERPIMAN, R.L. (1986). Inhibitory effect of warfarin on the metastasis of the PAIII prostatic adenocarcinoma in the rat. *J. Urol.*, **135**, 163–166.

O'MEARA, R.A.Q. (1958). Coagulative properties of cancers. *Irish Med. J.*, **394**, 474–479.

PANGASNAN, R.S., DEVEREUX, D., DECUNZO, L.P. & KARP, G.I. (1992). The production of a factor X activator by a methylcholanthrene-induced rat fibrosarcoma. *Thromb. Haemost.*, **68**, 407–412.

POLLARD, M. & LUCKERT, P.H. (1975). Transplantable metastasising prostate adenocarcinomas in rats. *J. Natl Cancer Inst.*, **54**, 643–649.

POLLARD, M. & LUCKERT, P.H. (1986). Production of autochthonous prostate cancer in Lobund–Wistar rats by treatment with N-Nitroso-N-methylurea and testosterone. *J. Natl Cancer Inst.*, **77**, 583–587.

PUSEY, M.L. & MENDE, T.J. (1985). Studies on the procoagulant activity of human amniotic fluid. 2. The role of factor VII. *Thromb. Res.*, **39**, 571–585.

SACK, G.H., LEVIN, J. & BELL, W.R. (1977). Trousseau's syndrome and other manifestations of chronic DIC in patients with neoplasms. *Medicine*, **56**, 1–37.

SZCZEPANSKI, M., BARDADIN, K., ZAWADZKI, J. & PYPNO, W. (1988). Procoagulant activity of gastric, colorectal, and renal cancer is factor VII-dependent. *J. Cancer Res. Clin. Oncol.*, **114**, 519–522.

TROUSSEAU, A. (1865). Phlegmasia alba dolens. In *Clinique Medicale de l'Hotel de Paris*, Anonymous (ed.) pp. 654–656. Balliere: Paris.

WEISS, H.J. (1975). Platelet physiology and abnormalities of platelet function. *N. Engl. J. Med.*, **293**, 531–541.

WHITMORE, W.F.J. (1990). Natural history of low stage prostate cancer and impact of early detection. *Urol. Clin. N. Am.*, **17**, 689–697.

WIJNGAARDS, G. & IMMERZEEL, J. (1977). Divalent cations and complex formation between tissue thromboplastin and factor VII. *Biochem. Biophys. Res. Commun.*, **77**, 658–664.

ZACHARSKI, L.R. & MEEHAN, K.R. (1993). Anticoagulants and cancer therapy. *Cancer J.*, **6**, 16–20.

ZACHARSKI, L.R., MEMOLI, V.A. & ROUSSEAU, S.M. (1986). Coagulation-cancer interaction in situ in renal cell carcinoma. *Blood*, **68**, 394–399.